# Culture-Dependent and Culture-Independent Methods in Evaluation of Emission of *Enterobacteriaceae* from Sewage to the Air and Surface Water

**DOI:** 10.1007/s11270-012-1171-z

**Published:** 2012-04-26

**Authors:** Ewa Korzeniewska, Monika Harnisz

**Affiliations:** Department of Environmental Microbiology, Faculty of Environmental Sciences and Fisheries, University of Warmia and Mazury in Olsztyn, Prawocheńskiego 1, 10-957 Olsztyn, Poland

**Keywords:** *Enterobacteriaceae*, Sewage, Bioaerosol, Water pollution, FISH, WWTP

## Abstract

The number of *Enterobacteriaceae*, with particular attention given to the presence of *Escherichia coli* and *Klebsiella pneumoniae*, was determined in hospital effluents and municipal wastewater after various stages of purification. The emission of these microorganisms to the ambient air near wastewater treatment plant (WWTP) facilities and to the river water, which is a receiver of the WWTP effluent, was also studied using fluorescence in situ hybridization (FISH) and cultivation methods. The number of *Enterobacteriaceae* determined by cultivation and fluorescence methods in different kinds of sewage sample ranged from 0.5 × 10^3^ to 2.9 × 10^6^ CFU/ml and from 2.2 × 10^5^ to 1.3 × 10^8^ cells/ml, respectively. Their removal rates during treatment processes were close to 99 %, but the number of these bacteria in the WWTP outflow was quite high and ranged from 5.9 × 10^3^ to 3.5 × 10^4^ CFU/ml and from 1.1 × 10^5^ to 6.1 × 10^5^ cells/ml, respectively. In the river water and the air samples, the number of *Enterobacteriaceae* was also high and ranged from 4.1 × 10^3^ to 7.9 × 10^3^ CFU/ml and from 3 to 458 CFU/m^3^, respectively. The numbers of these microorganisms obtained from fluorescence and cultivation methods were statistically and significantly correlated; however, the analysis of the studied samples indicated that the FISH method gave values up to 10^3^-fold times greater than those obtained by the cultivation method. From a sanitary point of view, this means that the number of viable fecal bacteria is systematically underestimated by traditional culture-based methods. Thus, the FISH proves to be a method that could be used to estimate bacterial load, particularly in air samples and less contaminated river water.

## Introduction

Wastewater treatment plants are recognized as being important sources of microbial pollution of water, soil, and atmospheric air and may pose a health risk to plant workers and the surrounding population (Douwes et al. 2001; Fracchia et al. 2006; Grisoli et al. 2009; Reinthaler et al. 2010; Korzeniewska 2011). Hospital and municipal wastewaters may contain pathogenic viruses, multidrug-resistant bacteria, yeasts, protozoa, and parasite eggs (Li et al. 2009; Yang et al. 2009; Chagas et al. 2011). *Enterobacteriaceae* bacteria, which are natural microbiota of human gastrointestinal tract, represent a large part of the bacterial community of sewage (Wéry et al. 2008; Korzeniewska et al. 2009). These bacteria, including *Escherichia coli*, are important indicator organisms for evaluating bacteriological safety of drinking water, recreational area, or food. The number of *Enterobacteriaceae* bacteria in hospital and wastewater treatment plant (WWTP) effluents is also an indicator of the degree of wastewater treatment efficiency (Filipkowska 2003; Espigares et al. 2006). The transfer of these microorganisms from WWTP to the environment takes place primarily through the outflow of treated wastewater directly into surface water reservoirs which are the receivers of these sewage. They penetrate to the air mainly during the mechanical (moving of raw sewage in the grit tanks) and biological (aeration of wastewater in the bioreactors) processes of sewage purification (Prażmo et al. 2003; Fracchia et al. 2006; Korzeniewska et al. 2008; Heinonen-Tanski et al. 2009). As bioaerosols might be a vehicle for the dissemination of human and animal pathogens from wastewater, the workers of WWTPs and inhabitants of their surroundings may be exposed to harmful influence of microorganisms from the air (Bünger et al. 2007; Liebers et al. 2008; Turner et al. 2008; Dungan et al. 2010; Liu et al. 2011, Hara et al. 2011). Many gram-negative bacteria produce lipopolysaccharides (LPS) as a part of the outer membrane of their cell wall. These potentially toxic LPS are also referred to as endotoxins and are released upon cell lysis. Some strains of *E. coli* generate verotoxins and heat-labile enterotoxins, which induce a strong response from properly working human immune systems (Bitzan et al. 1994; Mudrak and Kuehn 2010). Exposure to airborne endotoxins can cause chronic fatigue or/and acute fever and inflammatory reactions in the respiratory tract (Krajewski et al. 2004). Therefore, it seems to be important to determine emitted microorganisms from wastewater to air in a short time. Relatively rapid methods to enumerate coliform bacteria based on the activities of specific marker enzymes (beta-d-galactosidase for coliform bacteria and beta-d-glucuronidase for *E. coli*) were developed and used instead of traditional selective media based on lactose fermentation (Bonadonna et al. 2007). However, some noncoliform bacteria, such as *Aeromonas* spp. and *Vibrio* spp. showing beta-d-galactosidase activity, have caused false positive reaction in the coliform assay (Geissler et al. 2000). Fluorescence in situ hybridization (FISH) with rRNA-targeted probes is, among other things, a staining technique which allows phylogenetic identification of bacteria in mixed community without prior cultivation by means of epifluorescence microscopy (Amann et al. 1996). In theory, each ribosome within a bacterial cell, containing one copy, each of 5S, 16S, and 23S rRNA, is stained by one probe molecule during the hybridization procedure. The high numbers of ribosomes per cell provide a natural signal amplification system (Pernthaler et al. 2001). Highly specific to *Enterobacteriaceae*, 16S rDNA oligonucleotide probe allowed rapid and accurate detection of these bacteria. As a technique allowing simultaneous visualization, identification, enumeration, and localization of individual microbial cells, FISH is useful for many applications in all fields of microbiology (Bertaux et al. 2007). FISH enables the detection of not only culturable but also unculturable organisms and can therefore help to understand the actual microbial pollution of the environment (Moter and Göbel 2000; Wagner et al. 2003).

The aim of this study was to determine the real impact of the WWTP on the microbiological pollution of the surrounding environment with particular emphasis on the air at the WWTP area and the river water, which was the receiver of the WWTP effluent. In as much as traditional culture-dependent methods of quantification and identification of emitted microorganisms are limited by the inability to count uncultivated or nonviable bacteria, the FISH technique was used in quantitative and qualitative assessment of *Enterobacteriaceae* bacteria in this study.

## Materials and Methods

### Sampling Location and Sample Collection

The untreated sewage samples from effluents of three hospitals (H1, H2, and H3) and municipal sewage samples, including inflow, sewage from aeration tank (bioreactor), and outflow, were collected to sterile bottles from WWTP with the capacity of 2.64 × 10^4^ m^3^ per day. The hospital effluents comprise usually less than 2 % of the raw sewage influent to the WWTP (54, 328, and 49 m^3^ per day from hospitals H1, H2, and H3, respectively). Treated sewage samples were collected directly at the outlet of the WWTP, and water samples of Łyna River were collected 200 m for a tributary of the WWTP effluent to the river. Air samples were collected using impact method at two sampling sites, which were selected with respect to the highest expected emissions of the bioaerosol, wind velocity, and direction on the sampling days. To detect the total counts of *Enterobacteriaceae* bacteria in the air by cultivation method, total number of bacteria by 4′,6-diamidino-2-phenylindole (DAPI) staining, and number of *Enterobacteriaceae* by in situ hybridization FISH, air samples were collected with the MAS-100 *Eco* (Merck) using parallel two Petri dishes, each containing 30 ml of phosphate buffered saline (PBS) according to Pascual et al. (2001) with own modification. The 4,000 l of air samples was impacted onto the PBS, and afterwards, the PBS was transferred to sterile screw-capped tubes.

Sewage, air, and water samples were collected parallel during 13 months at approximately 8-week intervals. During the study, 42 of sewage samples, 14 of river water, and 14 of air samples were collected. All samples were transported refrigerated to the laboratory for immediate analysis.

### Determination of Environmental Parameters

The meteorological conditions like the speed and the direction of the wind and the temperature and humidity (measured by thermoanemometer and electronic hygrometers, respectively) of the air were measured parallel.

## Microbiological Methods

### Isolation of *Enterobacteriaceae* Bacteria from Sewage and Water Samples

For quantitative analysis by cultivation method, an amount of 1 ml of sewage/river water was diluted in 9 ml sterile PBS. In order to reduce the bacterial density, a decimal dilution series with PBS were prepared. Subsequently, 0.1 ml each of the well-homogenized solution was placed on Chromocult coliform agar (CCA) and incubated for 24 h at 35 ± 2°C.

### Isolation of *Enterobacteriaceae* Bacteria from Air Samples

Of the 60 ml PBS with air samples, 20 ml of PBS suspension was filtered through the polycarbonate filters with 0.22-μm pores (Millipore) to concentrate cells. Then, filters were placed on the surface of CCA medium into the Petri dishes. The Petri dishes with CCA and filters were incubated for 24 h at 35 ± 2°C. Following incubation, *Enterobacteriaceae* and *E. coli* colonies were counted basing on the color reaction of the CCA medium where they take on a celadon, purple, or blue color, and then, bacteria were analyzed for the production of cytochrome oxidase using 1 % tetramethyl-*p*-phenylenediamine solution. An oxidase-negative bacteria were identified as *Enterobacteriaceae*.

### Samples Fixation 

For FISH and DAPI quantification of bacteria, portions of 8 ml of each well-homogenized (homogenization by Silent Crusher, 7,000 rev/min, Heidolph) sewage sample and 40 ml of PBS with each air sample were fixed with a freshly prepared paraformaldehyde (PFA, pH 7.4) solution to a final concentration of 4 % (*v*/*v*) and stored for 1 h at room temperature. Then, the samples (parallel 1 ml of sewage–PFA solution and 1 ml of sewage–PFA solution diluted 1:10 with PBS;,50 ml of PBS with air sample and PFA solution) were filtered through white polycarbonate filters (pore size 0.2 μm, diameter 47 mm; type GTTP, Millipore) by using a gentle vacuum and cellulose nitrate support filters (pore size, 0.45 μm, Sartorius) to optimize the distribution of cells on the filters. Filters were subsequently washed twice with 10–20 ml of ultrapure water (MQ, Millipore), dried at room temperature, cut into 12 sections, and stored in Petri dishes at −20°C until further processing.

### FISH and Probe-Specific Cell Counts

Total bacterial numbers were determined after filtration of samples and staining filters with DAPI, final concentration 0.1 μg/ml (Pernthaler et al. 2001). Whole-cell in situ hybridization of sections from the polycarbonate filters was performed with the oligonucleotide probes EUB338 (Amann et al. 1990), GAM42a with competitor (Manz et al. 1992), ENT183 (Friedrich et al. 2003), ECO1167 (Neef et al. 1995), and KPN (Kempf et al. 2000), as described previously by Pernthaler et al. (2001). Aliquots of all samples were tested in parallel with the probe NON338 (Wallner et al. 1993), complementary to EUB338, in order to control nonspecific binding of the probes. The probe sequences, hybridization conditions, and references are given in Table 1. Oligonucleotides labeled with the cyanine dye Cy3 (red signal) were synthesized by Metabion (Martinsried, Germany). Filter sections were covered in mountant four parts of Citifluor (Agar Scientific, Essex, UK) and one part of VectaShield (Vector Laboratories, Burlingame, CA). Bacteria cells on the filter sections were observed with an epifluorescence microscope (BX61, Olympus) equipped with filter sets for DAPI and Cy3. Each microscopic field was first viewed with the CY3 filter set before switching to the DAPI filter set, to avoid bleaching of CY3 during the DAPI examination. For each sample and probe, more than 500 cells were enumerated; for the DAPI examination, between 500 and 1,000 DAPI-stained objects were evaluated per sample (with the exception of the air samples). All probe-specific cell counts were presented as the percentage of cells visualized by DAPI. The mean value and standard deviations were calculated from the counts of 20 randomly chosen fields on each filter section.

**Table 1:** Oligonucleotide probes used in this study

Probe	Specificity	Primer sequence 5′ → 3′	% FA^a^ in situ	Reference
EUB338	Bacteria	GCT GCC TCC CGT AGG AGT	35	Amann et al. (1990)
NON338	Control probe complementary to EUB338	ACT CCT ACG GGA GGC AGC	35	Wallner et al. (1993)
GAM42a	Gammaproteobacteria	GCC TTC CCA CTT CGT TT	35	Manz et al. (1992)
GAM42a’	Gamma competitor	GCC TTC CCA CAT CGT TT	35	Manz et al. (1992)
ENT183	*Enterobacteriaceae*	CTC TTT GGT CTT GCG ACG	20	Friedrich et al. (2003)
ECO1167	*E. coli*	GCA TAA GCG TCG CTG CCG	40	Neef et al. (1995)
KPN	*K. pneumoniae*	CCC TCT GAT GGG TAG GTT	30	Kempf et al. (2000)

^a^Percentage of formamide (FA) in in situ hybridization buffer

### Statistical Methods

The one-way ANOVA was used to evaluate whether the number of studied groups of bacteria detected in the sewage, river water, and air samples were dependent on the time of sampling. Moreover, the relationship between the number of studied microorganisms in hospital effluents, in municipal sewage after different steps of treatment and their incidence in air samples collected near grit chamber and aeration tank, and in the river water samples was evaluated. The relationship between the number of *Enterobacteriaceae* and *E. coli* bacteria isolated by cultivation and FISH methods was also analyzed. The relationship between the number of estimation by Spearman's correlation was used in this study. The tests were performed with the software STATISTICA 9.0 (StatSoft, Poland).

## Results and Discussion

### Number of *Enterobacteriaceae* Bacteria by Cultivation Method

The total number of *Enterobacteriaceae* and *E. coli* bacteria determined by cultivation method in different kind of sewage samples ranged from 0.5 × 10^3^ to 2.9 × 10^6^ CFU/ml and from 0.1 × 10^3^ to 1.3 × 10^5^ CFU/ml, respectively (Table 2). The highest numbers of these bacteria were determined in sewage samples collected from untreated sewage inflowing to WWTP and in sewage from aeration tank (bioreactor). Similar results were obtained by Korzeniewska et al. (2009), who stated the numbers of *Enterobacteriaceae* bacteria in wastewater from bioreactor in the amount of 2 × 10^5^–4 × 10^7^ CFU/ml. As Wéry et al. (2008) reported that *E. coli* bacteria in untreated and treated wastewater could range between up to 2.9 × 10^5^ and 8.6 × 10^1^ CFU/ml, respectively. In the present study, the number of *Enterobacteriaceae* and *E. coli* bacteria isolated by culture-based method from WWTP effluent reached values up to 2.1 × 10^4^ and 2.7 × 10^3^ CFU/ml, respectively. Their removal rates during treatment processes were close to 99 %, but the number of these bacteria in analyzed WWTP effluent was quite high and ranged from 5.9 × 10^3^ to 3.5 × 10^4^ CFU/ml and from 4.5 × 10^2^ to 6 × 10^3^ CFU/ml, respectively. In the river water samples, the number of *Enterobacteriaceae* and *E. coli* bacteria was also high and ranged from 4.1 × 10^3^ to 7.9 × 10^3^ CFU/ml and from 1 × 10^2^ to 3 × 10^3^ CFU/ml, respectively. According to many authors, the sites of pretreatment and the primary clarifiers, as well as those sites containing moving mechanical equipments for water aeration, are the steps with the highest emission of bioaerosols (Fracchia et al. 2006; Korzeniewska et al. 2008). The aeration system used in the biological process greatly affects the amount of bioaerosols generated (Sánchez-Monedero et al. 2008). In the presented study, higher numbers of *Enterobacteriaceae* and *E. coli* bacteria in the ambient air, ranging respectively from 3 to 458 CFU/m^3^ and from 1 to 30 CFU/m^3^, were more often observed in air samples collected near grit chamber than in air samples collected near aeration tank (bioreactor). The number of tested groups of bacteria in analyzed air samples collected by the method used in the present study was very close to the number of these bacteria in air samples collected parallel at the same sites and time using the impact method by Korzeniewska (own studies, unpublished data). She found that the number of *Enterobacteriacea*e and *E. coli* bacteria in the air samples ranged from 3 to 820 CFU/m^3^ and from 1 to 25 CFU/m^3^, respectively. Korzeniewska et al. (2009) and Filipkowska et al. (2000) observed the highest numbers of *Enterobacteriaceae* bacteria reaching values up to 5.0 × 10^2^ CFU/m^3^ near grit/grate chambers and 2.2 × 10^3^ CFU/m^3^ near aeration chambers, respectively. Similar results were found by Heinonen-Tanski et al. (2009), who observed the highest microbiological air contamination in pretreatment and aerated grit separation stages of wastewater treatment. This was attributed to aerosolization of microorganisms by mechanical handling or forced aeration.

**Table 2:** Number of *Enterobacteriaceae* and *E. coli* isolated by cultivation method and total DAPI cell counts and relative percentages of hybridized cells with specific probes

Sampling site (*n*)^a^	Number of *Enterobacteriaceae* (CFU × 10^3^)^b^ (mean ± SD)	Number of *E. coli* (CFU × 10^3^)^b^ (mean ± SD)	Absolute DAPI counts (cells/ml × 10^8^) (mean ± SD)	Fraction (%) of total cells (mean ± SD) detected with probe^c^				
				EUB338	GAM42a	ENT183	ECO1167	KPN
Hospital 1 (7)^a^	170 ± 100	3 ± 2	1.2 ± 0.9	74.8 ± 13	9.1 ± 5	7.3 ± 4	2.2 ± 1	1.7 ± 1
Hospital 2 (7)	100 ± 90	4 ± 3	1.3 ± 1.2	71.2 ± 12	7.2 ± 3	4.2 ± 7	1.4 ± 2	1.5 ± 2
Hospital 3 (7)	2730 ± 180	16 ± 15	1.4 ± 0.8	66.7 ± 5	4.7 ± 2	1.1 ± 1	0.2 ± 0.1	0.3 ± 0.1
Inflow sewage (7)	1910 ± 680	55 ± 42	3.5 ± 1.8	76.5 ± 9	8.3 ± 3	0.5 ± 0.1	0.2 ± 0.1	0.1 ± 0.1
Sewage in aeration tank (7)	690 ± 460	15 ± 14	73.7 ± 27	59.3 ± 13	12.1 ± 4.7	0.8 ± 0.5	0.5 ± 0.3	0.4 ± 0.5
Outflow (7)	21 ± 10	3 ± 2	0.5 ± 0.3	29.5 ± 13	17.1 ± 16	1.1 ± 1	0.3 ± 0.1	0.2 ± 0.1
River water (14)	6 ± 2	0.2 ± 0.1	0.05 ± 0.01	38.5 ± 17	19.1 ± 15	1.9 ± 1.1	0.4 ± 0.1	0.3 ± 0.1
Air near grit chamber (7)	0.29 ± 0.3	0.02 ± 0.01	0.03 ± 0.01	62.3 ± 19	48.2 ± 33	8.7 ± 9	8.0 ± 7	5.1 ± 4
Air near aeration tank (7)	0.04 ± 0.03	0.01 ± 0.01	0.02 ± 0.01	81.1 ± 15	33.8 ± 18	11.2 ± 11	7.0 ± 8	5.0 ± 9

*n* number of samples, *CFU* colony forming units, *SD* standard deviation

^a^In cultivation methods, the number of samples were performed in triplicate

^b^CFU on CCA medium by cultured method: in sewage and water—in colony forming units per milliliter, in air—in colony forming units per cubic meter

^c^Percent detection compared to DAPI; numbers have been corrected by subtracting NON338 counts

### DAPI Staining and FISH Results

Absolute DAPI counts in sewage samples ranged from 1.7 × 10^7^ cells/ml in sewage outflow from WWTP to 1.1 × 10^10^ cells/ml in sewage collected from aeration tank, while in the river water and air samples, counts of DAPI cells were from 1 × 10^2^- to 1 × 10^5^-fold lower, respectively (Table 1). The bacteria hybridized with probe EUB338, probe GAM42a, probe ENT183, probe ECO1167, and probe KPN respectively accounted for 65.1, 18.3, 4.6, 3.9, and 1.9 % of the total microorganisms from the DAPI staining (Table 2). Numbers of *Enterobacteriaceae*, *E. coli*, and *Klebsiella pneumoniae* were similar in sewage of hospital outflows and inflow to WWTP and ranged from 8.5 × 10^5^ to 7.4 × 10^6^ cells/ml (Table 2). The number of these groups of bacteria was highest in sewage from the aeration tank (bioreactor) and reached a value up to 1.3 × 10^8^, 8 × 10^7^, and 7.6 × 10^7^ cells/ml, respectively. Patentalakis et al. (2008), observed *E. coli* and *K. pneumoniae* in wastewater from aeration tank in the amount of 10^9^ cells/ml. Their results were about two orders of magnitude higher than those reported in this study. In the present study, the number of *Enterobacteriaceae*, *E. coli*, and *K. pneumoniae* in the air samples collected near aeration tank was slightly higher than in the air samples collected near grit chamber and ranged from 5 × 10^4^ to 6.2 × 10^5^ cells/m^3^ (Table 2). Patentalakis et al. (2008), observed these groups of microorganisms in the amount of 10^3^ cells/m^3^, which was about two orders lower than those reported in this study. This could be a result of the differences in the kind of treated sewage and in the applied wastewater aeration technologies.

### Comparisons of Bacterial Concentrations Obtained by FISH and Cultivation Methods and Variability in Their Number During Whole Time of Study

The number of the majority of studied microorganisms groups isolated from air samples was statistically and significantly correlated with the speed and the direction of the wind. The number of tested bacteria was not correlated with temperature and humidity of the air. There were also no statistically significant differences in their number, depending on the time of collecting of sewage, river water, or air samples. Higher number of these bacteria, however, was observed in spring and autumn. The numbers of *Enterobacteriaceae* and *E. coli* obtained from fluorescence and cultivation methods, were statistically and significantly correlated (Table 3). The analysis of studied samples indicated, nevertheless, that FISH method gave values ranging from two to four orders greater than those obtained by the cultivation method on CCA medium (Table 2). Rinsoz et al. (2008), who compared the number of mesophilic bacteria in air sampled at different sewage treatment plants from real-time PCR and epifluorescence methods, found that bacterial load was comparable. Their analysis indicates that these methods gave values 270- to 290-fold greater than those obtained by the “impaction on nutrient agar” method. Data in the present study suggest that the proportion of viable but nonculturable *Enterobacteriaceae* and *E. coli* increases in slightly less contaminated outflow, river water, and air samples. This can be probably explained by more stressful conditions in less contaminated environment. Similar results were obtained Garcia-Armisen and Servais (2004), who analyzed enumeration of viable *E. coli* in rivers and wastewaters by FISH and culture-based methods. They observed that the proportion of viable but noncultured *E. coli* detectable by the FISH technique was higher in less contaminated water. The results of the presented studies point out the limitations of culture-based methods to assess the quantity of airborne bacteria. It should be accepted that knowledge of the microbial diversity is incomplete, since people are not able to cultivate the vast majority (>90–99 %) of naturally occurring microorganisms using standard techniques (Amann et al. 1995). Therefore, the use of molecular methods seems to be necessary to estimate the spread of microorganisms from WWTPs to the environment and their emissions into the ecosystems.

**Table 3:** Statistic estimation by Spearman’s correlation between numbers of studied microorganisms determined by cultured method on CCA medium and fluorescence techniques (DAPI and FISH) in sewage, water, and air samples collected from different sites during the whole time of study

	Total number of *Enterobacteriaceae*	Number of *E. coli*
Number of *E. coli*	0.875*	
DAPI	0.850*	0.715*
EUB 338	0.860*	0.734*
GAM 42	0.817*	0.705*
ENT 183	0.708*	0.602*
ECO	0.490*	0.399*
KPN	0.401*	0.384*

*p < 0.05 (statistically significant correlation)

## Conclusions 

The results demonstrated that an epifluorescence microscopic FISH procedure can be used to enumerate viable *Enterobacteriaceae* not only in wastewater and river water but also in air samples. This method, in contrast to the culture-based methods, allows to obtain results within one or two working days and could be considered as a routine microbiological environmental analysis. Moreover, in the case of wastewater, it does not need multiple dilution of wastewater samples. The comparison of the number of cells detected by FISH method with a culture-based method used for the routine microbiological control of samples suggested the presence of a large proportion of viable *Enterobacteriaceae* bacteria not detected by the classic methods. This suggests a large underestimation of the abundance of *Enterobacteriaceae*, *E. coli*, and other groups of microorganisms in environmental samples. From a sanitary point of view, this means that the number of viable fecal bacteria is systematically underestimated by traditional culture-based methods, particularly in the air samples and less contaminated river water.
